# Beyond Dopamine Receptor Antagonism: New Targets for Schizophrenia Treatment and Prevention

**DOI:** 10.3390/ijms22094467

**Published:** 2021-04-25

**Authors:** Felipe V. Gomes, Anthony A. Grace

**Affiliations:** 1 Department of Pharmacology, Ribeirao Preto Medical School, University of Sao Paulo, Ribeirao Preto 01000-000, Brazil; gomesfv@usp.br; 2 Departments of Neuroscience, Psychiatry and Psychology, University of Pittsburgh, Pittsburgh, PA 15260, USA

**Keywords:** dopamine, psychosis, antipsychotics, hippocampus, parvalbumin, glutamate, stress

## Abstract

Treatment of schizophrenia (SCZ) historically relies on the use of antipsychotic drugs to treat psychosis, with all of the currently available antipsychotics acting through the antagonism of dopamine D2 receptors. Although antipsychotics reduce psychotic symptoms in many patients, they induce numerous undesirable effects and are not effective against negative and cognitive symptoms. These highlight the need to develop new drugs to treat SCZ. An advanced understanding of the circuitry of SCZ has pointed to pathological origins in the excitation/inhibition balance in regions such as the hippocampus, and restoring function in this region, particularly as a means to compensate for parvalbumin (PV) interneuron loss and resultant hippocampal hyperactivity, may be a more efficacious approach to relieve a broad range of SCZ symptoms. Other targets, such as cholinergic receptors and the trace amine-associated receptor 1 (TAAR1), have also shown some promise for the treatment of SCZ. Importantly, assessing efficacy of novel compounds must take into consideration treatment history of the patient, as preclinical studies suggest prior antipsychotic treatment may interfere with the efficacy of these novel agents. However, while novel therapeutic targets may be more effective in treating SCZ, a more effective approach would be to prevent the transition to SCZ in susceptible individuals. A focus on stress, which has been shown to be a predisposing factor in risk for SCZ, is a possible avenue that has shown promise in preclinical studies. Therefore, therapeutic approaches based on our current understanding of the circuitry of SCZ and its etiology are likely to enable development of more effective therapeutic interventions for this complex disorder.

## 1. Introduction

Schizophrenia (SCZ) is a severe psychiatric disorder that affects about 1% of the world population [1]. Patients afflicted with SCZ may experience a broad range of debilitating symptoms, with most of them categorized into three groups: positive, negative, and cognitive symptoms. Positive or psychotic symptoms include hallucinations, delusional ideas, and fragmentation of thinking. Negative symptoms include affective blunting, anhedonia, and social withdraw [2]. Cognitive symptoms are related to deficits in main cognitive domains such as working memory, attention, verbal learning and memory, and problem-solving [3,4].

Treatment of SCZ usually relies on symptom management, such as antipsychotic drugs to treat psychosis. Although antipsychotics reduce psychotic symptoms in many patients, some do not respond to treatment despite multiple trials of antipsychotic drugs [5]. Moreover, they may induce numerous undesirable effects that contribute to a high rate of treatment nonadherence [6]. In addition, although negative symptoms and cognitive impairments have been suggested as predictors of functional outcomes, there are currently no effective treatment options targeting them [3,7]. Together, these observations highlight the need to develop new drugs to treat SCZ.

## 2. Historical Perspective on the Mechanism of Action of the Currently Available Antipsychotics 

The initial breakthrough in the history of antipsychotics was the casual discovery of the effects of chlorpromazine in the 1950s. This dates back to the French surgeon Henry-Marie Laborit’s search for compounds capable of mitigating the “shock” due to excessive stress related to surgical procedures [8]. To mitigate this reaction, Laborit administered to patients a set of substances that he called “Lytic Cocktail” which contained, among other substances, the antihistaminic drug promethazine [8]. In search of new antihistaminic drugs, chlorpromazine, synthesized in 1950 by Paul Charpentier, was sent to Laborit. After administering this compound, Laborit observed that patients declared themselves “more relaxed and calmer”, showing “disinterest” in the face of the stress of the preoperative period without inducing marked sedation [8]. From these observations, it was suggested the potential use of chlorpromazine as a “tranquilizer”. The first clinical studies with this drug were carried out by Jean Delay and Pierre Deniker in Paris [9]. They resulted in strikingly positive findings, given that chlorpromazine significantly restored the patients’ quality of life, leading to wider use of this drug in psychiatry [10].

A few years after the introduction of chlorpromazine, other “first-generation” antipsychotics, with different chemical structures, such as haloperidol and fluphenazine, were introduced [10]. The advances in understanding their mechanisms of action came in the following decades. In 1963, Arvid Carlsson and Margit Lindqvist discovered that chlorpromazine and haloperidol increased monoamine metabolites in the mouse striatum that was interpreted as a compensatory effect to the blockade of blocking monoamine receptors [11]. It was quickly discovered that dopamine receptor blockade was the main mechanism of action. In the 1970s, independent studies from Philip Seeman and Solomon Snyder showed that all antipsychotics block postsynaptic dopamine D2 receptors. This was indicated by a highly significant correlation between the clinical potency of antipsychotics and their affinity for D2 receptors [12,13]. These findings guided research into the pathophysiology of SCZ and, together with the observation that drugs that potentiate dopamine neurotransmission, such as amphetamine, can mimic psychotic symptoms in healthy individuals [14], were the basis for the “dopamine hypothesis” of SCZ. This hypothesis proposes that a hyperdopaminergic state drives psychotic symptoms [15]. Recent neuroimaging studies have indeed shown an increased presynaptic dopamine function in striatal regions of SCZ patients who respond to antipsychotics [16,17]. These changes were found to be correlated with psychotic symptoms severity [18]. 

While D2 receptor antagonism accounts for the therapeutic effect of “first-generation” antipsychotics, this mechanism also results in severe side effects, such as extrapyramidal motor effects (parkinsonism), tardive dyskinesia, and hyperprolactinemia. The induction of extrapyramidal effects is so striking a characteristic of first-generation antipsychotics that it was initially thought of as a prerequisite for the therapeutic activity of these drugs [19]. This idea made a certain “candidate” antipsychotic, clozapine, be viewed with skepticism, since it did not induce extrapyramidal effects. Interest in clozapine was further reduced after the observation that it could induce agranulocytosis [19], which occurs in about 1% of patients.

Ironically, clozapine would become the “gold standard” for antipsychotics. In a classical clinical study, carried out in 1988 by Herbert Meltzer and colleagues, clozapine was found to have a unique antipsychotic efficacy by attenuating positive symptoms without causing motor side effects [20]. Clozapine was also somewhat effective against the negative symptoms [20]. These findings had marked implications. First, the influential hypothesis that extrapyramidal effects would be a prerequisite for antipsychotic activity was undermined. Second, this study revived interest in clozapine. Once rejected, this drug became the prototype in the search for new “second-generation” antipsychotics. 

Several hypotheses to explain the unique efficacy of clozapine were proposed, including the one proposed by Herbert Meltzer, which is based on a favorable ratio between the antagonism of serotonin 5-HT2A and D2 dopamine receptors [21]. While the blockade of D2 receptors would be necessary for the antipsychotic effect, the blockade of 5-HT2A receptors would be a protective factor against extrapyramidal side effects. This model produced a series of second-generation antipsychotics, such as risperidone, ziprasidone, quetiapine, and olanzapine, which displayed a lower propensity to induce motor side effects at therapeutic doses than first-generation antipsychotics but with no better efficacy. However, second-generation antipsychotics are associated with off-target receptor activity-related effects on metabolic changes and weight gain [22].

Despite the importance of Meltzer’s proposal, other hypotheses have been developed to differentiate antipsychotics based, for example, on how antipsychotics occupy D2 receptors. Neuroimaging studies with SCZ patients under treatment carried out by Farde et al. [23,24] evaluated the percentage of D2 receptors occupied in the striatum by therapeutic doses of several antipsychotics. Therapeutic doses of clozapine, which did not induce extrapyramidal effects, resulted in about 65–70% of D2 receptors’ occupancy. On the other hand, first-generation antipsychotics, which induce extrapyramidal effects, caused more than 80% of receptors’ occupancy at therapeutic doses. Therefore, it was suggested that occupation of D2 receptors above 65% seems necessary for the therapeutic effect, while an occupation exceeding 80% results in extrapyramidal effects. That is, the occupation of receptors and, therefore, the dose necessary of second-generation antipsychotics for the therapeutic effect is, in general, less than that required to induce motor side effects. Later, a pharmacological mechanism to explain these differences was proposed by Shitij Kapur and Philip Seeman in which second-generation antipsychotics dissociated more readily from the D2 receptor than first-generation antipsychotics [25,26]. Thus, contrary to Meltzer’s proposal on the affinity ratio between 5-HT2A and D2 receptors [21], Kapur’s dissociation hypothesis predicts that a second-generation antipsychotic can be selective for the D2 receptor [27] —as it is the case for amisulpride [28]. Instead, the requirement was that it dissociate from the receptor more readily and be displaced by dopamine when released. This would allow dopamine to bind to the receptor to produce its physiological function, for example, in regions associated with motor control, without inducing extrapyramidal effects [27].

The previous discussions were based on the fact that antipsychotics act as D2 receptor antagonists. However, the interpretation of the works by Farde et al. [23,24], that extrapyramidal effects occur when there is a striatal D2 receptor occupation higher than 80%, no longer covers all antipsychotics, particularly with regard to partial agonists. For example, aripiprazole, which acts as a D2 receptor partial agonist [29,30], has a therapeutic effect with 85–95% striatal D2 receptor occupation without producing motor side effects [31]. Some referred to aripiprazole and other D2 receptor partial agonists, such as brexpiprazole and cariprazine, as “third-generation” antipsychotics [32]. As partial agonists, these drugs activate the D2 receptor to a lower degree than dopamine. Therefore, they are thought to stabilize dopamine neurotransmission by reducing excessive striatal D2 receptor stimulation through a functional antagonism of excessive dopamine release to relieve the positive symptoms in SCZ. However, while the other antipsychotics significantly attenuate the activation of D2 receptors, aripiprazole, as a partial agonist, can activate these receptors although less effectively than dopamine itself. This preserves the function of the extrapyramidal system since the drug partially fulfills the function of the endogenous ligand (dopamine). In addition, while D2 receptor antagonists, by blocking both presynaptic and postsynaptic D2 receptors, results in overexcitation-induced depolarization block which leads to a broad reduction in dopamine neuron activity and responsivity [33], aripiprazole does not induce depolarization block [34] and instead may act as an agonist on presynaptic D2 receptors to downregulate dopamine neuron activity [34]. Furthermore, its lower intrinsic activity at the receptor than dopamine may simultaneously explain a potential antagonist-like effect at postsynaptic D2 receptors in hyperdopaminergic states [35].

More recently, the U.S. Food and Drug Administration approved lumateperone for the treatment of SCZ. This compound has a unique mechanism of action by simultaneously modulating dopamine, serotonin, and glutamate neurotransmission [36]. In contrast to most first- and second-generation antipsychotics, that are both presynaptic and postsynaptic D2 receptor antagonists, and aripiprazole and related compounds, that are both presynaptic and postsynaptic D2 receptor partial agonists, lumateperone is reported to act as presynaptic partial agonist and a postsynaptic antagonist at D2 receptors [36]. Interestingly, the improvement in SCZ symptoms induced by lumateperone [37] was associated with approximately 40% striatal D2 receptor occupancy [38], which is a substantially lower occupancy at an efficacious dose than most currently available antipsychotics that exhibit 60% to 80% D2 receptor occupancy [23,24]. This may be due to a contributing attenuation of dopamine neuron firing/dopamine release via its presynaptic inhibitory action, as has been shown for aripiprazole [34]. Moreover, in addition to its action at D2 receptors, lumateperone act as a potent serotonin 5-HT2A receptor antagonist and indirectly enhances glutamate neurotransmission downstream of dopamine D1 receptor activation [36], which may lead to increases in the activity of both NMDA and AMPA glutamate receptors. Despite its unique pharmacological profile, since lumateperone, similar to most second-generation antipsychotics, has antagonist effects at the D2 and 5-HT2A receptors, it is not clear how unique of a mode of action it represents [39].

Overall, the antagonism of D2 receptors is thought to be the primary mechanism of all currently available antipsychotics on positive symptoms of SCZ. Despite advances in recent years, effective treatment of SCZ remains an issue. Except for clozapine, which has a unique efficacy but indicated only for treatment-resistant psychosis due to the potential induction of agranulocytosis, current antipsychotic drugs have similar efficacy on positive symptoms [40,41]. Moreover, antipsychotics typically do not provide relief from negative and cognitive symptoms [3,7] and up to 25% of SCZ patients do not respond even for positive symptoms [5]. Additional challenges include nonadherence to treatment and adverse effects, especially extrapyramidal motor effects and metabolic dysregulation [6,42]. Therefore, there is an urgent need for new antipsychotic drugs with better efficacy and tolerability.

## 3. New Targets to Treat SCZ

Although current antipsychotics directly target the dopamine system, there is little evidence for dysfunction within dopaminergic neurons themselves [43]. Instead, the aberrant dopamine transmission and associated SCZ symptoms have been proposed as a consequence of disruption in afferent brain regions that regulate the dopamine system, mainly cortical and hippocampal regions [43,44,45]. Hence, treatment at the site of pathology could be a more effective therapeutic avenue than current antipsychotics that target D2 receptors.

**Figure 1:**
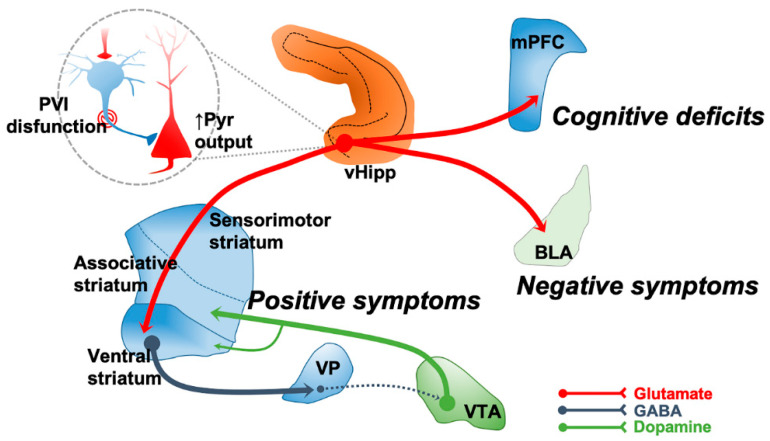
The anterior limbic hippocampus in humans, which is homologous to the ventral hippocampus (vHipp) of the rodent, is proposed to be hyperactive and dysrhythmic in SCZ due to a decreased PV interneuron (PVI) inhibition of pyramidal (Pyr) neurons. This is thought to lead, through a ventral striatum-ventral pallidum (VP) pathway, to an overdrive in the activity of VTA dopamine neurons that project to the associative striatum. The resulting striatal hyperdopaminergic state has been linked to the positive symptoms of SCZ. Additionally, a hyperactive hippocampus can also interfere with the function of other circuits. For instance, disruption of prefrontal cortex (PFC) and basolateral amygdala could potentially lead to cognitive deficits and interfere with emotional responses leading to negative symptoms, respectively. Therefore, a hyperactive dysrhythmic limbic hippocampus potentially disrupts multiple circuits and could contribute to the three main symptom clusters of SCZ.

Some of the more consistent alterations in SCZ are associated with a dysregulation of excitatory and inhibitory neurotransmission in cortical and hippocampal regions. Decreased GABAergic signaling is among the most robust postmortem pathological changes observed in SCZ [46]. These deficits are largely restricted to GABAergic interneurons containing the calcium-binding protein parvalbumin (PV) [47,48]. These neurons synapse on the cell body and/or the axon initial segment of glutamatergic pyramidal neurons regulating their output. Furthermore, it is likely that a functional loss of perisomatic targeting PV interneurons results not only in a decreased inhibitory control over pyramidal neuron activity, but also disruption in coordinated rhythmic oscillatory activity across a broad neural network [45,49]. Oscillatory activity in general, and gamma oscillations (30–80 Hz) in particular, represent the functional state and coordinated activity within neuronal networks [50,51]. Gamma oscillations are implicated in cognitive processes and rely on intact PV interneuron function [50]. In SCZ, dysfunction of PV interneurons has been linked to disturbances in gamma oscillations that are thought to contribute to impaired cognition [45,52,53]. In addition, abnormal excitatory–inhibitory balance in cortical and hippocampal regions may lead to dysregulation of the midbrain dopamine system activity [43,44,45]. For example, in the hippocampus a functional loss of PV interneurons has been associated with hippocampal hyperactivity that is proposed to underlie the hyperdopaminergic state observed in SCZ [54,55]. A hyperactive hippocampus can also interfere with the function of other circuits leading to cognitive deficits and negatives symptoms (Figure 1). Thus, targeting excitatory–inhibitory balance may alleviate positive, negative, and cognitive symptoms of SCZ [54]. Therefore, one potential approach for treating a broad range of SCZ symptoms is to modulate abnormalities in glutamate and GABA neurotransmission, as will be discussed below. Other targets, such as cholinergic receptors and the trace amine-associated receptor 1 (TAAR1), will also be discussed.

### 3.1. Targeting Excitatory–Inhibitory Dysregulation

**Figure 2:**
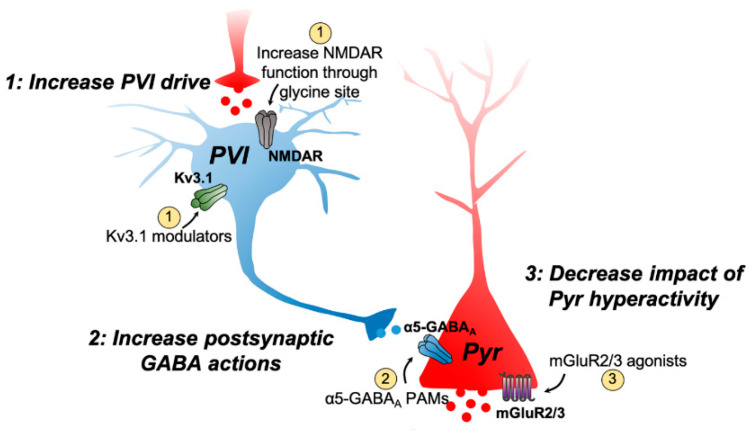
Targeting the dysregulation of excitatory–inhibitory balance in SCZ include compounds that may compensate for the functional loss of PV interneurons (PVI) and attenuate the potentially increased activity of pyramidal (Pyr) neurons as well as the resulting increase in glutamate release. A hypofunction of NMDA receptors on PVI is proposed to underlie SCZ symptoms. (**1**) Compounds that facilitate NMDA receptor activity without triggering excitotoxicity, such as compounds that act through the glycine site, have the potential to increase PVI drive. A further approach that could normalize the functional loss of PVI is the modulation of Kv3.1 potassium channels on these cells. These channels play an important role in regulating PVI activity by allowing these cells to fire at high frequency. (**2**) Another approach that could compensate for decreases in PVI functionality is to increase postsynaptic GABA actions. One target that has shown some promise is the GABA_A_ receptor containing the α5 subtype (α5-GABA_A_). (**3**) A decreased PVI inhibition of pyramidal neurons leads to a greater glutamate release. Therefore, another target is the use of agents that decrease presynaptic glutamate release, such as group II metabotropic glutamate receptor (mGluR2/3) agonists have attracted great interest as a novel treatment for SCZ.

Opportunities for the development of drugs targeting the dysregulation of excitatory–inhibitory balance include compounds that may compensate for the functional loss of PV interneurons and attenuate the potentially increased activity of pyramidal neurons as well as the resulting increase in glutamate release. It has been suggested that a functional deficit in PV interneurons in SCZ may result from either a loss of PV neurons in the hippocampus [56] or loss of PV activity in the hippocampus and PFC due to hypofunction of NMDA receptors on these cells [57]. This, along with other evidence indicating an NMDA receptor hypofunction in SCZ, has supported efforts to develop drugs that facilitate NMDA receptor activity without triggering excitotoxicity (Figure 2). Initial clinical studies using the endogenous co-agonist of NMDA receptors glycine or D-serine as an add-on treatment to antipsychotics indicated some beneficial effects on positive, negative, and cognitive symptoms in SCZ [58,59,60,61,62]; however, this was not replicated in larger studies [63,64]. Moreover, the use of these compounds may be limited by tolerability issues. Compounds that increase glycine availability by inhibiting the glycine transporter 1 (GlyT1) have also been investigated. The most extensively studied GlyT1 inhibitor was bitopertin from Roche. Despite some promise in preclinical [65] and initial clinical studies as an adjunct therapy [66], bitopertin failed in both phase 2 and phase 3 clinical trials as either a monotherapy or adjunct therapy [67,68,69]. Similar to GlyT1 inhibitors, increasing D-serine levels through the inhibition of its metabolizing enzyme D-amino acid oxidase (DAAO) also has the potential to increase NMDA receptor function in SCZ [70]. Two small clinical trials showed that sodium benzoate, a DAAO inhibitor, as adjunctive therapy improved various symptom domains in patients with chronic SCZ [71,72]. However, these findings have yet to be replicated in larger trials. In addition, the DAAO inhibitors currently available present some features that may limit their use, such as poor bioavailability and poor ability to cross the blood–brain barrier [73].

A further approach that could potentially normalize the functional loss of PV interneurons would be the modulation of Kv3.1 potassium channels on these cells. The Kv3.1 channel is part of the family of Kv3-type voltage-gated potassium channels (Kv3.1-Kv3.4) that have fast-spiking properties [74]. Kv3.1 channels are abundantly expressed in PV interneurons and play an important role in regulating their activity by allowing these cells to fire at high frequency and, thus, enabling the synchronized activity of pyramidal neurons and generation of gamma oscillations [75,76], which is dramatically impaired in SCZ [53]. Therefore, the modulation of these channels could potentially normalize the impaired activity of these interneurons in SCZ (Figure 2). Some experimental compounds that act as positive modulators of Kv3.1 channels showed the ability to rescue the fast-spiking phenotype of parvalbumin-positive-fast-spiking interneurons following an impairment of their firing capacity and behavioral impairments in animal models of SCZ based on NMDA receptor hypofunction [77,78]. More recently, a preliminary study showed that a Kv3.1 modulator reduced the increases in cortical blood oxygen level-dependent (BOLD) signal in healthy volunteers induced by the NMDA receptor antagonist ketamine [79]. However, studies evaluating the effects of Kv3.1 modulators in SCZ patients have not as yet been reported.

Another approach that could compensate for decreases in PV interneuron functionality is to increase GABA neurotransmission. Broad action GABA modulators, such as benzodiazepines, are problematic due to their sedative actions, risk of dependency and lack of efficacy in improving psychotic symptoms in chronic SCZ patients [80], but others, with a more localized action, have been studied for their potential therapeutic value in SCZ. One target that has shown some promise is the GABA_A_ receptor containing the α5 subtype (α5-GABA_A_) [81], which is highly expressed in limbic brain regions, mainly in the hippocampus and to a lesser extent in the neocortex [82,83,84]. A proposed function of α5-GABA_A_ receptors is the tonic regulation of inhibitory inputs to pyramidal neurons, coordinating spike timing of these neurons and balancing excitation [85,86,87]. Of particular interest is the involvement of the α5-GABA_A_ receptor present on pyramidal neurons regulating GABA inputs arising from perisomatic targeting PV-expressing interneurons (Figure 2). Preclinical studies have supported the potential use of α5-GABA_A_ receptors positive allosteric modulator (PAM) to treat SCZ. For example, in the MAM model, which is characterized by a marked hyperdopaminergic state driven by ventral hippocampus hyperactivity [88], an α5-GABA_A_ PAM reduced hippocampal hyperactivity and normalized the increased dopamine neuron population activity in the ventral tegmental area and locomotor response to amphetamine in MAM rats when administered either systemically or infused into the ventral hippocampus [89]. In addition, the overexpression of the α5-GABA_A_ receptor within the ventral hippocampus normalized ventral hippocampus hyperactivity and downstream alterations in ventral tegmental area (VTA) dopamine neuron function as well as cognitive disruption in the MAM model [90]. These findings suggest that the α5-GABA_A_ receptor may be an effective target for normalizing hippocampal activity in SCZ, but it has yet to be tested in patients.

A decreased PV interneuron inhibition of pyramidal neurons proposed to underlie SCZ symptoms leads to a greater glutamate release. This is consistent with findings showing elevated glutamate levels in some brain regions of SCZ patients [91]. Thus, compounds that decrease presynaptic glutamate release may show some promise. In this sense, group II metabotropic glutamate receptor (mGluR2/3) agonists have attracted great interest as a novel treatment for SCZ. mGluR2/3 are expressed in limbic brain regions and localized presynaptically on glutamatergic terminals to negatively regulate glutamate release (Figure 2) [92]. Preclinical research produced extensive support for mGluR2/3 agonists [93,94,95]. The mGluR2/3 agonist from Eli Lilly, pomaglumetad, was shown to reduce hippocampal hyperactivity in the MAM model, resulting in the downstream normalization of VTA dopamine neuron population activity [96]. In SCZ patients, pomaglumetad produced beneficial effects on both positive and negative symptoms in early clinical trials as a monotherapy in a phase 2 clinical trial [97]. However, in subsequent trials, it failed to show efficacy as a monotherapy or adjunct therapy [98,99,100,101]. Later analyses of trial data suggested that certain patients may respond better to pomaglumetad, particularly those who were in the earlier phases of illness [102]. Although additional trials are needed to understand why certain groups may respond better to pomaglumetad, these findings indicate that the efficacy of pomaglumetad in SCZ may depend on treatment history and disease progression.

It is worth noting that dissociation between preclinical and early phase clinical studies and the longer-term multiple site trials may be impacted by prior antipsychotics use. Whereas in preclinical studies drugs are commonly tested on antipsychotic-naïve animals, in clinical trials the drugs are tested on SCZ patients that had received chronic antipsychotic therapy, often for decades, prior to a 1–2 week washout period. Indeed, the induction of D2 receptor supersensitivity by prior treatment with current antipsychotics may interfere with the efficacy of novel target agents [103]. This may account for the pomaglumetad actions on early-stage patients that have not had a long course of antipsychotic exposure [102].

### 3.2. Targeting Cholinergic Receptors

Several lines of evidence point to dysfunction in the cholinergic system in SCZ. Among these is the observation that an estimated 60–80% of SCZ patients are smokers, with a pattern of more heavy and intense smoking in smokers with SCZ compared to the general population [104,105]. Although no mechanistic explanation has yet been established, it is proposed that nicotine, the primary reinforcing component of tobacco, relieves SCZ symptoms (“self-medication” hypotheses) and/or SCZ may confer enhanced rewarding effects from nicotine [106]. These hypotheses were recently tested in the MAM model. While a nicotine self-administration scheme did not lead to increased reinforcement [107], nicotine administration may have therapeutic actions since it normalized behavioral and neurophysiological perturbations in MAM rats [108].

Muscarinic and nicotinic acetylcholine receptors (mAChRs and nAChRs) are emerging as targets for developing novel treatments for SCZ. There is a growing interest in the study of α7-nAChR and mAChR M1 and M4 as potential targets. Preclinical and early clinical studies have provided evidence that compounds that activate mAChR M1 and M4 and α7-nAChR produce antipsychotic-like effects and/or cognitive enhancement in animal models and the treatment of positive and cognitive symptoms in SCZ patients. However, while early attempts to develop selective mAChR and nAChR agonists provided exciting preliminary findings, these compounds have ultimately failed in clinical development due to a lack of subtype selectivity and subsequent dose-limiting adverse effects [109]. We recently found that α7-nAChR agonists administered systemically or into the ventral hippocampus counteracted the hyperdopaminergic state in the MAM model [110]. This indicates that the ventral hippocampus can be a site of action for these compounds. In the hippocampus, the activation of α7-nAChRs on interneurons increases the frequency of inhibitory postsynaptic currents in pyramidal neurons [111,112], which may normalize disruptions in the excitatory-inhibitory balance. Thus, an increase in the activity of GABAergic interneurons in the ventral hippocampus followed by the administration of α7-nAChR agonists could attenuate the hyperactivity of pyramidal neurons and the resulting enhanced VTA dopamine system activity in the MAM model [113]. In humans, agonists of α7-nAChRs have been evaluated as an adjunct therapy to antipsychotics in clinical trials of SCZ patients. However, findings have been disappointing, with no significant effects on cognitive impairment or negative symptoms [114]. 

Regarding mAChRs, multiple lines of evidence point to mAChR M1 and M4 as potential targets for SCZ. Xanomeline, an mAChR M1/M4 preferring agonist, showed efficacy in animal models of SCZ [115,116,117] and human trials for psychosis and cognitive function of Alzheimer’s disease patients [118] and total symptoms of SCZ in treatment-refractory patients [119]. However, in both studies high rates of pro-cholinergic side effects, such as nausea, vomiting, and diarrhea, were observed, leading to the discontinuation of xanomeline as a monotherapy. To overcome these side effects, xanomeline was combined with trospium chloride, a peripherally restricted pan-muscarinic antagonist approved for overactive bladder [120] that may induce anticholinergic-like side effects limited to anticholinergic effects, such as constipation and dry mouth, and reduces peripheral cholinergic effects of xanomeline. In a phase 2 clinical trial, xanomeline/trospium demonstrated significant antipsychotic efficacy with an improved safety profile, lowering rates of cholinergic adverse events. It is still unclear if both mAChR M1 and M4 agonism are required for xanomeline efficacy [121]. Several mechanisms have been proposed to explain how agonists of mAChR M4 could counteract a hyperdopaminergic state. The activation of mAChR M4 on cholinergic interneurons in the striatum reduces local cholinergic tone within the striatum, which reduces striatal dopamine levels [122,123]. Similar outcomes were found by Foster and colleagues, where the activation of mAChR M4 receptors on D1 receptor-spiny projection neurons may increase the release of the endocannabinoid 2-arachidonoylglycerol that, through the activation of cannabinoid CB2 receptors located in presynaptic terminals of dopamine neurons, leads to sustained inhibition of dopamine release [124]. In addition, the activation of presynaptic mAChR M4 located in laterodorsal tegmental nuclei cholinergic neurons projecting to the VTA reduces local cholinergic tone, which may modulate dopamine neuron activity [117].

### 3.3. Targeting Trace Amine-Associated Receptor 1 (TAAR1)

Trace amine-associated receptor 1 (TAAR1) is a G-protein-coupled receptor activated by endogenous trace amines that are structurally related to monoaminergic neurotransmitters. The expression of TAAR1 was reported in several brain regions, such as the prefrontal cortex, striatum, amygdala, nucleus accumbens, and ventral tegmental area [125]. It is proposed that the activation of TAAR1 modulates presynaptic dopamine synthesis capacity [126], which may produce antipsychotic-like effects. Moreover, TAAR1 may alter D2 receptor-mediated signaling through the formation of heterodimers [127].

In mice, SEP-363856, a TAAR1 agonist developed by Sunovion Pharmaceuticals, reduced dopamine synthesis capacity induced by repeated treatment with ketamine [128]. SEP-363856 was also found to inhibit neuronal firing and decrease excitability in the ventral tegmental area [129]. In addition to TAAR1, SEP-363856 also acts as a 5-HT1A receptor agonist [129]. Recent clinical trials have evaluated SEP-363856 for SCZ. In a phase-II, randomized, double-blind, placebo-controlled 4-week, SEP-363856 was superior to placebo for reducing both positive and negative symptoms of SCZ without inducing side effects of current antipsychotics [130]. Phase 3 clinical trials are ongoing. Clinical trials investigating the effects of ralmitaront (RO6889450), a TAAR1 partial agonist, in SCZ are also ongoing. 

## 4. Preventive Interventions in SCZ

SCZ is a neurodevelopmental disorder more commonly diagnosed in late adolescence and early adulthood. In some patients, a “clinical high-risk” or “prodromal” state, characterized by functional decline and sub-clinical psychotic symptoms, precedes SCZ [1]. While novel therapeutic targets may be more effective in treating SCZ, a more effective approach would be to prevent the transition from a high-risk state to SCZ. Several have targeted this early phase to find strategies to prevent progression to the full-blown disorder, which occurs in 20–35% of cases [131].

Although its causes are not completely known, it is thought that SCZ arises from interactions between genetic predisposition and socio-environmental risk factors. Indeed, it is known that genetics alone does not determine SCZ since the concordance rate for SCZ in identical twins is around 50% despite identical genetic makeup and around one-third of SCZ is not familial [1]. In addition, it is known that socio-environmental factors can increase the probability of psychosis in susceptible individuals [132] with childhood trauma playing a major role [133,134].

One socioenvironmental risk factor that has been associated with SCZ development is stress. Importantly, stress before or during adolescence is particularly impactful as a risk factor for SCZ [135]. Thus, in studies of children at risk for SCZ, the individuals that showed the greatest stress response tended to convert to SCZ later in life [136]. Furthermore, childhood stress or trauma is associated with SCZ onset in late adolescence/early adulthood [137] and the severity of positive symptoms [138].

Similar to the clinics [139], an increased responsivity to stress has observed in several animal models of SCZ based on neurodevelopmental disruption [140,141]. For example, in the MAM model, MAM rats show abnormal stress reactivity and heightened anxiety prepubertally [140,142] prior to the full expression of SCZ-related changes [143,144]. This enhanced responsivity to stress is proposed to lead to PV interneuron damage in the ventral hippocampus and, consequently, to hippocampal hyperactivity and dopamine system overdrive [135]. Furthermore, studies have shown that exposure of normal rats to adolescent stress can lead at adulthood to changes similar to those found in the adult MAM rat [144], including a loss of PV interneurons and increased activity of pyramidal neurons in the ventral hippocampus, basolateral amygdala hyperactivity, and increased activity of the VTA dopamine system [145,146,147]. On the other hand, the same stress protocol applied to adult animals resulted in transient depression-related changes [145].

If exposure to stress during critical periods of development, such as childhood and adolescence, may lead to SCZ, then decreasing stress during these periods could circumvent the damage that leads to the emergence of SCZ later in life. Indeed, we found that administering the anti-anxiety drug diazepam at a dose sufficient to attenuate anxiety and reverse amygdala hyperactivity decreased PV interneuron loss in the ventral hippocampus and prevented the emergence of the hyperdopaminergic state in the adult MAM rat [142,148,149]. Benzodiazepines are not a feasible prophylactic option for several reasons, including dependence and tolerance and the low incidence of transition to SCZ, but these findings suggest that reducing the deleterious impact of stress during adolescence, particularly in individuals with a family history and showing high anxiety levels, may be an effective approach to prevent the emergence of SCZ. It is likely that other stress-relieving interventions, such as cognitive remediation, cognitive behavioral therapy, and psychosocial therapies, will also be effective due to their potential to mitigate stress reactivity, which could have beneficial effects in protecting circuits from disruption. More recently, we found that prepubertal environmental enrichment was sufficient to prevent dopamine hyperresponsivity in adult MAM rats through normalizing ventral hippocampal pyramidal neuron activity but without reducing anxiety-like changes [150].

Periods before and during adolescence are proposed to be a time at which PV neurons are of higher susceptibility to stress-induced damage. Studies have shown that the PV interneurons play a unique role in neuronal systems development. In the prefrontal cortex and hippocampus, PV interneurons continue to mature until late adolescence/early adulthood [151,152]. PV neurons exhibit a substantial amount of plasticity early in life, with glutamatergic synapses forming and being removed as the organism learns to deal with environmental contingencies. However, this plasticity comes at a price, in that the PV neurons are highly vulnerable to stressors at this time point. Thus, oxidative stress, glutamate drive, high-frequency firing, could all contribute to pathology and cell death [135]. This vulnerability continues until the closure of the critical period by the formation of perineuronal nets, a glycosaminoglycan matrix sheath that surrounds mainly PV interneurons and stabilizes glutamatergic inputs to end the plastic phase, but also protect PV interneurons from metabolic and oxidative damage [153]. For this reason, the timing of the stressor may be critical in determining the outcome.

**Figure 3:**
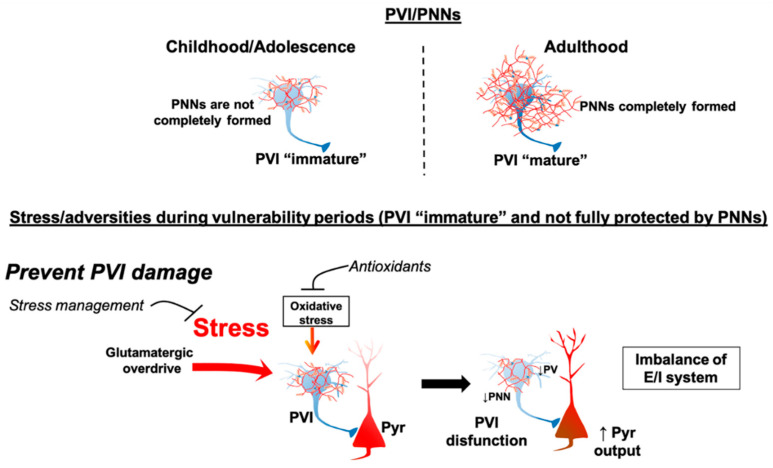
It is proposed that during childhood/adolescence PV interneurons (PVI) are not “mature” and are not yet protected by perineuronal nets (PNNs), a glycosaminoglycan matrix sheath that surrounds PVI to end the plastic phase, but also protect PVI from metabolic and oxidative damage. Thus, during periods when PNNs are not yet fully formed, PVI are more vulnerable to stress-induced damage. This vulnerability continues until adulthood. During adolescence, exposure to stress can increase oxidative stress and cause aberrant excitation onto PVIs leading to PVI damage/loss. This, in turn, results in deficits in the excitatory/inhibitory (E/I) balance producing circuit deficits that lead to SCZ-related changes. Therefore, treatments that mitigate the impact of stress, through stress management approaches and/or attenuation of oxidative stress with antioxidants, may protect PVI from damage and potentially prevent SCZ development.

It is well-known that stressful events lead to oxidative stress, which is characterized by a disturbance in the balance between the production of reactive oxygen species and antioxidant defenses. A redox dysregulation is proposed to play a role in the development of SCZ [154]. Increased oxidative stress at the end of adolescence/early adulthood has been implicated in PV interneuron loss in the ventral hippocampus [155] and disruption in the formation of perineuronal nets [153]. Based on these evidences and supported by preclinical studies, some have suggested using antioxidants, N-acetyl-cysteine (NAC), and sulforaphane, as a potential strategy to prevent SCZ. In rodents, juvenile and adolescent treatment with NAC prevented the reduction of prefrontal PV interneuron activity as well as electrophysiological and behavioral deficits in a SCZ model based on ventral hippocampus neonatal lesion [156]. In humans, NAC supplementation for 6 months was found to mitigate some symptoms in early psychosis patients [157,158]. Overall, treatments that mitigate the impact of stress and protect PV interneurons may effectively prevent SCZ development (Figure 3).

## 5. Conclusions

SCZ has historically been treated via administration of D2 antagonists; a therapeutic approach that emerged as a result of a serendipitous finding unrelated to SCZ. An advanced understanding of the circuitry of SCZ has pointed to pathological origins in the excitation/inhibition balance in regions such as the hippocampus, and restoring function in this region, particularly as a means to compensate for PV interneuron loss and resultant hippocampal hyperactivity, may be a more efficacious approach. However, initial positive results from preclinical studies and early clinical trials of compounds that act on this system have not been successful. One caveat is that these compounds were tested on chronic SCZ patients that had been exposed to D2 receptor antagonist antipsychotic drugs for many years before being withdrawn for 1–2 weeks prior to administering the test compound. While this may be sufficient to clear the drug from the system, it will not restore the system to normal. Preclinical studies suggest that antipsychotic drug-induced postsynaptic D2 receptor supersensitivity can interfere with the action of novel compounds. Thus, clinical trials should control for the prior medication history in terms of the antipsychotic drug used (“first”, “second” or “third-generation” partial agonist antipsychotic) and duration of treatment. The problem of supersensitivity may be circumvented by transition from an antipsychotic that does not cause supersensitivity, such as aripiprazole which does not induce dopamine neuron depolarization block and supersensitivity [34]. In addition, to correlate the impact of new drugs on preclinical indices such as dopamine system activity, amygdala activity, and hippocampal firing normalization with clinical outcomes has the potential to provide valuable insight into SCZ treatment. 

Another potentially more important route of investigation relates to prevention of the transition to SCZ in susceptible individuals. A focus on stress, which has been shown to be a predisposing factor in risk for SCZ [136], is a possible avenue that has shown promise in preclinical studies. Therefore, therapeutic approaches based on our current understanding of the circuitry of SCZ and its etiology are likely to enable development of more effective therapeutic interventions for this heretofore difficult disorder.
